# Attitudes of nursing students towards ageism and associated factors

**DOI:** 10.17533/udea.iee.v42n3e14

**Published:** 2024-10-30

**Authors:** Jaedson Capitó de Santana, Julio César Menezes de Peixoto Filho, Rose Dayanne da Silva Araújo, Jack Roberto Silva Fhon, Fábia Maria de Lima

**Affiliations:** 1 Nurse; Master in Nursing. Email: Jaedson.capito@upe.br. Corresponding author. https://orcid.org/0000-0001-7315-9635 Universidade de Pernambuco Brazil Jaedson.capito@upe.br; 2 Nurse. Email: julio.peixoto@upe.br. https://orcid.org/0000-0003-0786-4445 Universidade de Pernambuco Brazil julio.peixoto@upe.br; 3 Nurse. Email: rose.araujo@upe.br. https://orcid.org/0009-0008-6362-5768 Universidade de Pernambuco Brazil rose.araujo@upe.br; 4 Nurse; PhD in Nursing; PhD Professor at the School of Nursing of the University of São Paulo in the Department of Medical Surgical Nursing. Email: betofhon@usp.br. https://orcid.org/0000-0002-1880-4379 Universidade de São Paulo School of Nursing University of São Paulo Department of Medical Surgical Nursing Brazil betofhon@usp.br; 5 Nurse; PhD in Neuropsychiatry and Behavioral Sciences; PhD Professor at the Nossa Senhora das Graças College of Nursing, University of Pernambuco. E-mail: fabia.lima@upe.br. https://orcid.org/0000-0001-9992-6556 Universidade de Pernambuco Nossa Senhora das Graças College of Nursing University of Pernambuco Brazil fabia.lima@upe.br; 6 University of Pernambuco (UPE) Universidade de Pernambuco University of Pernambuco Brazil; 7 University of São Paulo, (USP), Brazil. Universidade de São Paulo University of São Paulo Brazil

**Keywords:** older adults, ageism, attitudes, aging, nursing students., ancianos, edadismo, actitudes, envejecimiento, estudiantes de enfermería., idoso, idadismo, atitudes, envelhecimento, estudantes de enfermagem.

## Abstract

**Objective.:**

To evaluate the associated factors on ageism in nursing students from a public university in Recife-PE.

**Methods.:**

This is a cross-sectional, quantitative, analytical study, whose participants (*n*=215) were students of the bachelor's degree in Nursing at a public university in Recife, Pernambuco, Brazil. Data regarding responses to a sociodemographic questionnaire and Fraboni Ageism Scale were analyzed.

**Results.:**

The characteristics that predominated among the participants were: female (83.3%), age between 17-20 years (41.9%), single (92.1%), did not have a scholarship (83.5%), lived with parents and/or siblings (60.9%), had no child (98.1%), did not live with the older adult (74.4%), and was attending the first year of under graduation (28.4%). On the Fraboni Scale, the score was 36.74 out of 84 possible points for ageism, being by domain: 11.55 points for Avoidance, 11.54 points for Antilocution, and 10.05 points for Discrimination**.**

**Conclusion.:**

Among the nursing students surveyed, ageism was associated with the male gender, younger age, not being a scholarship holder and living alone. Education and training aimed at caring for the older adults are effective tools to challenge prejudice related to aging.

## Resumen

**Objetivo.:**

Evaluar los factores asociados al edadismo entre los estudiantes de enfermería de una universidad pública de Recife-PE.

**Métodos.:**

Se trata de un estudio transversal, cuantitativo, analítico, cuyos participantes (*n*=215) eran estudiantes del curso de Licenciatura en Enfermería de una universidad pública de Recife, Pernambuco, Brasil. Se analizaron datos de un cuestionario sociodemográfico y de la Escala de Edad de Fraboni.

**Resultados.:**

Las características que prevalecieron entre los estudiantes fueron: sexo femenino (83.3%), tener entre 17 y 20 años (41.9%), ser soltero (92.1%), no tener beca de estudios (83.5%), vivir con los padres y/o hermanos (60.9%), no tener hijos (98.1%), no vivir con personas mayores (74.4%) y cursar el primer año de licenciatura (28.4%). En la Escala de Fraboni, la puntuación fue de 36,74 puntos sobre 84 posibles para el edadismo, y por dominios: 11.55 puntos para la Evitación, 11.54 puntos para la Antilocución y 10.05 puntos para la Discriminación.

**Conclusión.:**

Entre los estudiantes de enfermería encuestados, el edadismo se asoció al género masculino, a la menor edad, a no estar becado y a vivir solo. La educación y la formación orientadas al cuidado de las personas mayores son herramientas eficaces para hacer frente a los prejuicios relacionados con la edad

## Resumo

**Objetivo.:**

Avaliar os fatores associados sobre o ageísmo nos estudantes de enfermagem de uma universidade pública do Recife-PE.

**Métodos.:**

Trata-se de um estudo transversal, quantitativo, analítico, cujos participantes (*n*=215) foram discentes do curso de bacharelado em Enfermagem de uma universidade pública de Recife, Pernambuco, Brasil. Foram analisados dados referentes a respostas de um questionário sociodemográfico e Escala de Fraboni de Ageísmo.

**Resultados.:**

As características que predominaram entre os participantes foram: sexo feminino (83.3%), idade entre 17-20 anos (41.9%), solteiro (92.1%), não possuía bolsa estudantil (83.5%), morava com pais e/ou irmãos (60.9%), não tinha filho (98.1%), não convivia com idoso (74.4%), e estava cursando o primeiro ano de graduação (28.4%). Na Escala de Fraboni, a pontuação foi de 36,74 de 84 pontos possíveis para o ageísmo, sendo por domínio: 11.55 pontos para o Evitamento, 11.54 pontos para Antilocução, e 10.05 pontos para Discriminação**.**

**Conclusão.:**

Entre os estudantes de enfermagem pesquisados, o ageísmo foi associado ao gênero masculino, à idade mais jovem, ao fato de não ser bolsista e de morar sozinho. A educação e o treinamento voltado ao cuidado em idosos são ferramentas eficazes para desafiar o preconceito relacionado ao envelhecimento.

## Introduction

Population aging is a global reality that has been intensifying in recent years. It is a natural and lifelong process that, although universal, is not uniform. There are morphological, functional, biochemical, physical and mental changes in it, which occur in a different way and speed for both sexes.^1^ This phenomenon is shaped by the relationships one has with the social and physical environments in which the person lives throughout life, varying according to some characteristics, such as personal, family, sex and ethnic characteristics.^2^According to the United Nations, the number of old adults aged 60 and over was 202 million in 1950, increased to 1.1 billion in 2020, and is expected to reach 3.1 billion by 2100.^3^ In Brazil, population aging was highlighted by the total number of people aged 60 or over in the country in the last census of 2022, pointing out 32,113,490 older people, corresponding to 15.6% of the population.^4^ With the advent of the phenomenon of population aging, ageism, a term that emerged with Robert Buttler ^5^ to refer to discrimination against individuals based on their age, which can cause harm, disadvantages and injustices and erode solidarity between different generations, has become more common, even if not explicit.^6^

Ageism can manifest itself at the individual, institutional, social or political level, encompassing the different social units, person, family, community and society, through prejudices and erroneous stereotypes, producing discriminatory attitudes and behaviors towards the older person.^7^ With the increase in life expectancy, older people and very older people have been nursing clients. Identifying and understanding the ageism present among health professionals is pertinent, since age prejudice causes them to attribute the physiological complications experienced by the older adult to "unfortunate consequences of aging", thus leading to inadequate care, with tendencies to offer or provide inadequate care and treatment to the older adult compared to the younger ones.^8^^,^^9^

Care for the older adults can be inhibited by ageism among health professionals, including acts and behaviors, such as speaking low to the older adults and/or providing unequal care to the older adults compared to the younger ones, in addition to minimizing interest in the area of aging: studies show that nursing students have a decreased desire to work in the area of gerontology.^10^ In addition, nursing students expressed age behaviors, such as talking slower or louder with the older adults, and cementing characteristics of how an older person should be and act.^11^ A study conducted in Jordan with 290 nursing students, the data indicate that most of them reported insufficient knowledge and had discriminatory attitudes and behaviors related to age. In addition, a significant difference was found between the students of the latter year and the former in relation to knowledge about aging.^12^

Strategies to reduce ageism include international laws and public policies such as the Madrid Political Declaration and International Plan of Action on Ageing, the European Union Directive on equal treatment in employment, the Protocol on the Rights of Older Persons in Africa and the Inter-American Convention on the Protection of the Human Rights of Older Persons.^6^ In addition, studies indicate that educational interventions are among the most efficient strategies for reducing ageism.^10^^-^^13^ An educational intervention was conducted in Mazandaran province (Iran) with primary, secondary, high school and university students. The intervention consisted of ten workshops addressing issues about human aging, and included lectures, films, brochures, discussions, and conversations with older people. Before the intervention, it was found that primary, secondary and high school students were less ageist in relation to university students, according to the Fraboni Ageism Scale (FSA). The intervention lowered ageism scores in all groups of students. The study also found that ageism was more prevalent among nursing and medical students when compared to students in other courses.^13^ Providing education and training to nursing students in order to know and reduce age-related prejudice is important in the process of care and biopsychosocial well-being of the older adults, which in the future will have an impact on the nursing care of the older adults. Thus, the objective of this study was to evaluate the factors associated with ageism in nursing students at a public university in Recife-PE.

## Methods

This is a quantitative cross-sectional study that is part of a multicenter study entitled “Attitudes and perceptions in nursing students: Brazil and Peru” carried out at the Nursing College Nossa Senhora das Graças of University of Pernambuco - FENSG/UPE, with headquarters and jurisdiction in the city of Recife-PE-Brazil. FENSG is a 77-year-old higher education institution that trains bachelors in Nursing, masters and PhD in Nursing. The nursing course is expected to last five years (10 terms), with 60 vacancies per entry in each semester. 

The sample size was estimated considering the number of 476 students enrolled in the academic semester of 2022.2, with a sampling error of 5% and a confidence level of 95%, indicating the need to collect at least 149 students. However, the selection of students was for convenience, where all had the same condition to participate as long as they completed the instrument within the stipulated deadline, and the risk of bias was not controlled, since all eligible students were invited to participate in the study. All 215 students who started completing the instrument completed it. 

To participate in the study, students had to meet the following inclusion criteria: be regularly enrolled in the nursing course in the 2022-2 semester. Students who were absent from the course due to withdrawal and/or illness were excluded from the research. The instruments used were the sociodemographic profile (age, sex, marital status, and year of study, paid work, student and family income, people with whom they live; lives with the older adult) and the Fraboni Ageism Scale was used to assess ageism. It evaluates explicit ageism through cognitive and affective aspects of prejudice from three levels (domains): antilocution, avoidance and discrimination.^14^ The Scale, validated for Brazilian Portuguese, presented a Cronbach's Alpha of α = 0.88 divided into three factors: separation (10 items, α = 0.85); stereotype (5 items, α = 0.78); and affective attitudes (6 items, α = 0.68). The scale consists of 21 questions, and the response scale is a four-point Likert type with response categories: 1=strongly disagree, 2=disagree, 3=agree and 4= strongly agree.^15^ The higher the scores, the greater the person's ageism. Each domain has seven items. It is important to note that questions B16 to B21 require differentiated attention in relation to the others, since the results in the Agree or Disagree indexes reflect the opposite, as they denote positive attitudes towards aging. Thus, their scores are inverted, so that they result in scores compatible with the others.

Data collection was carried out online through the Web-based Survey and made available to students via institutional e-mail and later, for greater adherence through the social network WhatsApp between January and May 2023. Upon accessing the research link, students were directed to the Informed Consent Form (ICF), where they could read and accept or not participate in the study. Acceptance or not to participate in the study was automatically recorded in the database.

The data obtained through the instruments were stored in a database in the Google Drive tool, which was exported to the *SPSS* software, where the analysis was performed.

To characterize the personal and clinical profile, habits and experience of abuse of the patients evaluated, the percentage frequencies were calculated and the respective frequency distributions were constructed. The Fraboni ageism scale measures were calculated in order to determine the domains with the highest level of ageism. To compare the mean of the total ageism score, avoidance score, discrimination score, antilocution score, and the ageism score in the organizational context, between the profiles of the students evaluated, the means and standard deviation of the scores were calculated. Each domain and total score of the Fraboni scale was analyzed using Pearson's correlation and Student's t-test. Multiple linear regression was used to identify the association between the domains and the total of the scale with the participants' sociodemographic variables. All statistical tests were considered significant when the p-value was equal to or less than 0.05.

The research was, in the form of a subproject, approved by the Research Ethics Committee under registration - CAAE 53589221.9.2001.5192 and opinion number 5,428,778. 

## Results

Of the population of 476 nursing students eligible for the research, there were 215 participants. There was no record of dropouts. Most students were female (83.3%), aged 17 to 20 years (41.9%), and single (92.1%), without a scholarship (83.5%), lived with their parents and siblings (60.9%), had no child (98.1%), did not live with the older adult (74.4%) and were attending the first year of under graduation (28.4%). Of the students who had some contact with the older adult, this was the grandfather or grandmother (87.3%). The correlation between the domains of the Fraboni scale and the total of the scale, and a correlation was identified between the antilocution domain and the year of under graduation of the participant (*p*=0.036).

Table 1 shows that the Avoidance domain was related to sex (*p*=0.018, higher in men), scholarship holder (*p*=0.042) and living with the older adult (*p*=0.005). The discrimination domain was also related to sex (*p*=0.050, higher in men), scholarship holders (*p*=0.011) and those who live with the older adult (p=0.032). In the same table, in the antilocution domain there was a relationship with sex (p=0.015, higher in men) and scholarship holder (*p*=0.046). Finally, in the comparison of means of the total scale, called ageism, there was a relationship with sex (*p*=0.023, higher in men), scholarship holder (*p*=0.015) and living with the older adult (*p*=0.019).

**Table 1:** Comparison of means between the domains and total of the Fraboni scale with the sociodemographic variables of the nursing student, Recife-PE, 2023

Domain	Variable	Category	*n*	Mean	SD	95% CI		p-value
Avoidance	Sex	Female	179	11.15	3.29	10.68	11.64	0.018
		Male	36	13.56	5.00	12.02	15.24	
	Marital status	Single	198	11.52	3.66	11.02	12.04	
		Married	14	12.00	4.72	10.17	15.27	0.922
		Divorced	3	12.00	4.36	6.51	15.10	
	Scholarship holder	No	180	11.77	3.72	11.24	12.33	0.042
		Yes	35	10.46	3.63	9.40	11.80	
	Lives with the older adult	No	160	11.23	3.92	10.65	11.87	0.005
		Yes	55	12.49	2.94	11.72	13.26	
Discrimination	Sex	Female	179	9.74	2.94	9.33	10.18	0.050
		Male	36	11.58	4.41	10.25	13.10	
	Marital status	Married	14	10.86	4.02	9.06	13.17	0.693
		Divorced	3	11.33	5.03	6.17	15.55	
		Single	198	9.97	3.22	9.54	10.44	
	Scholarship holder	No	180	10.29	3.31	9.83	10.80	0.011
		Yes	35	8.80	2.93	7.92	9.84	
	Lives with the older adult	No	160	9.86	3.49	9.35	10.44	0.032
		Yes	55	10.58	2.57	9.92	11.26	
Antilocution	Sex	Female	179	11.26	3.40	10.77	11.76	0.015
		Male	36	12.94	4.45	11.50	14.36	
	Marital status	Married	14	11.93	4.12	9.93	14.09	0.854
		Divorced	3	10.67	3.22	6.69	12.98	
		Single	198	11.53	3.62	11.03	12.04	
	Scholarship holder	No	180	11.77	3.54	11.27	12.30	0.046
		Yes	35	10.34	3.93	9.12	11.69	
	Lives with the older adult	No	160	11.35	3.78	10.78	11.95	0.101
		Yes	55	12.09	3.15	11.25	12.90	
Ageism	Sex	Female	179	35.65	9.48	34.29	37.06	0.023
		Male	36	42.11	14.64	37.51	46.94	
	Marital Status	Married	14	38.86	13.03	33.14	46.51	0.845
		Divorced	3	37.67	13.80	20.82	47.70	
		Single	198	36.57	10.59	35.14	38.08	
	Scholarship holder	No	180	37.50	10.59	36.00	39.09	0.015
		Yes	35	32.80	10.85	29.48	36.59	
	Lives with the older adult	No	160	35.93	11.32	34.26	37.76	0.019
Yes	55	39,07	8,55	36,70	41,19

SD = Standard Deviation; 95% CI = 95% confidence interval; p-value = statistical significance

The Hierarchical Linear Regression model was used to test predictors against domains and ageism. Table 2 shows that the Avoidance domain was associated with male gender (*p*<0.001), age (*p*=0.011) and not being a scholarship holder (*p*=0.036). On the other hand, in the Discrimination domain, it was associated with male gender (p=0.002), age (*p*=0.017), being single (*p*=0.036), not being a scholarship holder (*p*=0.017), and living alone (*p*=0.036).

The antilocution domain was associated with age (*p*=0.008), study time (*p*=0.003), not being a scholarship holder (*p*=0.048) and living alone (*p*=0.005). In the Fraboni Scale total model, called ageism, an association was identified with male gender (*p*=0.002), age (*p*=0.007), not being a scholarship holder (*p*=0.018) and living alone *(p*=0.012).

**Tabela 2:** Association between the domains of the Fraboni scale with the sociodemographic variables of nursing students. Recife-PE. 2023

Domain / variables	B	CI 95% (min/max)	***p-*value**
Avoidance			
Gender			
Female	-		
Male	2.869	1.288, 4.449	<0.001
Age	-0.298	-0.526, -0.070	0.011
Scholarship holder			
No	-		
Yes	-1.589	-3.074, -0.104	0.036
Discrimination			
Gender			
Female	-		
Male	2.218	0.812, 3.624	0.002
Age	-0.248	-0.451, -0.045	0.017
Marital status			
Married	-		
Divorced	-2.309	-9.136, 4.517	0.505
Single	-5.275	-10.207, -0.342	0.036
Scholarship holder			
No	-		
Yes	-1.615	-2.936, -0.294	0.017
Lives with the older adult			
With my parents and siblings	-		
Family and grandparents	1.052	-2.144, 4.249	0.516
Alone	1.926	0.125, 3.728	0.036
Only with grandparents	-0.830	-3.955, 2.295	0.601
Husband/Wife	-3.351	-8.703, 2.000	0.218
With friends /student house	-2.245	-4.978, 0.488	0.107
Antilocution			
Age	-0.294	-0.508, -0.079	0.008
Study time	0.295	0.104, 0.487	0.003
Scholarship holder			
No	-		
Yes	-1.410	-2.805, -0.015	0.048
Lives with the older adult			
With my parents and siblings	-		
Family and grandparents	0.910	-2.466, 4.285	0.595
Alone	2.713	0.811, 4.615	0.005
Only with grandparents	0.372	-2.928, 3.671	0.824
Husband/Wife	-2.094	-7.746, 3.557	0.465
With friends /student house	-2.729	-5.614, 0.157	0.064
Ageism			
Gender			
Female	-		
Male	7.218	2.707, 11.729	0.002
Age	-0.909	-1.560, -0.257	0.007
Scholarship holder			
No	-		
Yes	-5.119	-9.357, -0.881	0.018
Lives with the older adult			
With my parents and siblings	-		
Family and grandparents	3.738	-6.518, 13.994	0.473
Alone	7.403	1.623, 13.183	0.012
Only with grandparents	0.345	-9.681, 10.371	0.946
Husband/Wife	-3.714	-20.885, 13.456	0.670
With friends /student house	-7.230	-15.998, 1.538	0.105

## Discussion

In this research, the factors associated with ageism in nursing students were younger age, male gender, less course time, not living with the older adult, lower income and not receiving an academic scholarship. The sociodemographic data of the sample reveal an unequal distribution regarding sex, proportional to the national rate, in which according to the 2022 University Education Census, 57.7% of students enrolled in university education were women.^16^ The UPE Nursing course has a higher number of female students enrolled than the national mean (83.6%), also consistent with the distribution of the population by sex, both at the municipal, state and national levels, in which the female sex always prevails.^17^ This unequal proportion is related to the historical female predominance of the profession, despite the gradual increase in males pointed out in the literature.^18^

With regard to the age group of this study, the course is formed by young students aged 17 to 20, mostly single and without children. These results reflect the entry of these increasingly younger students into university education, as a consequence of factors such as the demands of the current economic market, improvements in access to university education, but also possible family and social demands to start under graduation immediately after high school. In addition, the marital status and number of children of most students in this study coincides with studies carried out in São Paulo and Rio de Janeiro, which justify this variable to probable influences of the course curriculum because it requires full dedication, and irregularity of the distribution of internships between the morning, afternoon and evening shifts, making family commitments difficult, and remaining in the labor market to obtain gains for family income.^19^ It is also observed that most participants did not receive financial support grants offered by the university. The literature points out that not receiving a scholarship can have repercussions on the dropout of many students throughout their academic lives, both because the student seeks extra income and does not get due to the dedication that the course requires, as well as the lack of financial supply to meet the demands required by the course itself, such as clothing, books and instruments.^20^ Allied to this is the fact that many students come from cities far from the capital or even from other states, with those responsible for the student bearing costs such as lodging, food and transportation.

In the analysis of the present study, it was found that there is a significant negative relationship between age prejudice scores and chronological age, where younger respondents tend to be more prejudiced in relation to those with more years. Such an association was already expected, since as people get older, they tend to become more critical of ageist attitudes.^21^ With regard to gender, the study points to a greater association between males and ageism. Also, a study carried out among nursing students and nursing professionals trained in Sweden and Greece showed that males and young age (less than 25 years) are important risk factors for the prediction of ageism in relation to the older adults, and that this difference in attitudes between men and women is possibly explained by the greater degree of empathy of women.^22^

Considering the correlation of course time (year of under graduation) and ageism, it was found that the shorter the time of under graduation course, the greater the ageism. In fact, the literature elucidates that exposure to geriatric/gerontological content in health courses improves the perception of attitudes towards the older adults ^23^ However, in the nursing course, knowledge about these subjects, and curricular internship practices aimed at the older adults, occur mainly from the 7^th^ semester of the course, which may explain this correlation of ageism and year of under graduation. A study that included 57 countries and estimated the global prevalence of ageism in the older adults and explored the possible explanatory factors revealed that the effect of schooling was more pronounced in relation to ageism than younger age or males.^24^ However, among the participants of this study, the correlation between sex-ageism and age-ageism was higher than between the latter variable and ageism.

Study participants who live with an older person demonstrated less ageist attitudes than those who do not. Studies reveal that good quality contact is generally the most reliable predictor of prejudice reduction than simple one-off contact. Thus, the simple sporadic connection with older adults may not be enough to minimize generational prejudice.^25^ A systematic review revealed that better quality contact, both with older adults in general and especially with grandparents and other older family members, reduces ageism.^26^ Another study conducted in 25 European Union countries found that people who reported having intergenerational friendships tended to be less ageist, applying to both younger and older adults,^27^ which reinforces the idea that the undergraduate nursing course should include opportunities for interaction with the older adult, especially among those who do not have quality intergenerational interactions.

Discussing the findings of this study on income and receipt or non-receipt of university scholarships correlated with ageism is challenging, since there are few studies that analyze the correlation of these variables. In general, it was evidenced that people who live with lower income and without academic scholarship tend to be more ageists. The analysis by Officer, et al.^24^ based on data from 57 countries found that 39% of survey participants in low- and lower-middle-income countries had highly ageist attitudes. To find that low income and ageism are associated is alarming, since about half of the world's population (48.3%) lives in low- and lower-middle-income countries (9.3% in low-income countries and 39% in lower-middle-income countries) ^28^. On the other hand, according to the study by Officer, et al.^24^, lower prevalence rates were present in higher income countries, such as Australia, Poland and Japan, where 69% of participants from high-income countries had lower rates of ageist attitudes when compared to 18% of participants in low- and lower-middle-income countries.

The literature points out that professionals with ageist attitudes tend to label older people as inflexible, unproductive, lonely, sick, senile, depressive, fragile and without energy.^29^ In addition, while the presence of ageism interferes with the way the health professional understands the aging process, he can attribute symptoms such as sadness and pain to age, making it difficult to recognize pathological needs and processes in older adults.^30^ Gould, Dupuis-Blanchard, and MacLennan^31^ conducted a qualitative study to explore attitudes toward the older adults among Canadian nursing students, where 20 students participated in the study. It was revealed that, even though students had positive reactions when caring for older patients, they saw gerontological nursing as a non-prestigious and non-valued area. The researchers concluded that additional nursing education about caring for patients in the context of aging was needed.

Within the context of ageism among university students, the literature is congruent in stating that the contact and interaction of the student with the older adult in the different contexts of under graduation is considered essential for the development of positive attitudes and skills for the care of these people.^6^ Nursing students participating in the study by Gould et al.^31^, when asked whether attitudes towards older adults have changed since the beginning of the course, considered that contact interventions with older patients was the key to the development of positive attitudes towards the older population in general. 

Educational interventions aimed at teaching about aging allow us to provide accurate information and examples that combat ageist stereotypes, dispelling misconceptions about this age group, teaching skills and knowledge, and allowing people to consciously reconsider and update their beliefs, feelings and behaviors, thus reducing ageism in the university environment.^30^ These interventions include, for example, role-plays, simulations, lectures, virtual reality, intergenerational contact, student internships and mentoring, both focused on the aging process, and which may be integrated into the curriculum of university education courses. Such educational interventions are effective in reducing ageism and the price seems to be affordable.^6^

Study limitations. As this is a study in which only one course from a university was analyzed, the results do not necessarily reflect the reality of all nursing courses and students in Brazil and the world. It is also important to note that, as the statistical analysis worked with convenience of samples, the restrictions on the generalization of these results are reinforced. The evidence obtained in this research can be extrapolated and explored in other contexts, in other courses or universities. In addition, the collection period took place during remote activities at the university due to the COVID-19 pandemic. There is evidence in the literature that the aforementioned disease has amplified harmful attitudes towards stereotypes, prejudices and discrimination based on age.^6^ Thus, there is a possibility of a negative bias in the scores of the variables of interest.

Conclusion From the research conducted, it can be seen that the ageism in the negative perspective of the FSA was more often associated with the male sex, not being a scholarship holder, not living with the older adult, not having children, and the initial semesters of under graduation; while the positive items of the FSA were more often associated with age and being a scholarship holder. Education and training aimed at caring for the older adults are effective and important tools to challenge prejudice related to aging. As a strategy for university education, nursing study programs should be integrated with a realistic and stimulating view of older adult care, providing the training of qualified nursing professionals with attitudes, knowledge and skills based on scientific evidence, offering gerontological care for a society that is increasingly aging.
